# Genome-Wide DNA Methylation Analysis and Epigenetic Variations Associated with Congenital Aortic Valve Stenosis (AVS)

**DOI:** 10.1371/journal.pone.0154010

**Published:** 2016-05-06

**Authors:** Uppala Radhakrishna, Samet Albayrak, Zeynep Alpay-Savasan, Amna Zeb, Onur Turkoglu, Paul Sobolewski, Ray O. Bahado-Singh

**Affiliations:** 1 Department of Obstetrics and Gynecology, Oakland University William Beaumont School of Medicine, Royal Oak, Michigan, United States of America; 2 Department of Obstetrics and Gynecology, Wayne State University School of Medicine, Detroit, Michigan, United States of America; South Texas Veterans Health Care System and University Health Science Center San Antonio, UNITED STATES

## Abstract

Congenital heart defect (CHD) is the most common cause of death from congenital anomaly. Among several candidate epigenetic mechanisms, DNA methylation may play an important role in the etiology of CHDs. We conducted a genome-wide DNA methylation analysis using an Illumina Infinium 450k human methylation assay in a cohort of 24 newborns who had aortic valve stenosis (AVS), with gestational-age matched controls. The study identified significantly-altered CpG methylation at 59 sites in 52 genes in AVS subjects as compared to controls (either hypermethylated or demethylated). Gene Ontology analysis identified biological processes and functions for these genes including positive regulation of receptor-mediated endocytosis. Consistent with prior clinical data, the molecular function categories as determined using DAVID identified low-density lipoprotein receptor binding, lipoprotein receptor binding and identical protein binding to be over-represented in the AVS group. A significant epigenetic change in the *APOA5* and *PCSK9* genes known to be involved in AVS was also observed. A large number CpG methylation sites individually demonstrated good to excellent diagnostic accuracy for the prediction of AVS status, thus raising possibility of molecular screening markers for this disorder. Using epigenetic analysis we were able to identify genes significantly involved in the pathogenesis of AVS.

## Introduction

Congenital heart defect (CHD) is the most common type of birth defect, and affects close to 1% of all live births [1, 2]. Over 50% of children born with CHD will have at least one invasive surgery in their lifetime, while many will have multiple surgeries. In the US, approximately 20–30% of all infants with CHD have valve defects [3, 4].

The etiology of CHD is multifactorial and complex. Approximately 30% of children with a chromosomal abnormality have associated CHD [5]; however, a majority of CHD cases have no identifiable genetic abnormality. Families with autosomal dominant [6], recessive [7] and X-linked [8] CHD have been reported; however, in the majority of cases genetic mechanisms have not heretofore been identified. It is postulated that sporadic CHD, which accounts for around 80% of all cases, arises from an interaction of genetic and environmental factors [9]. Gestational diabetes, dietary deficiency, medication use, maternal age, cigarette smoking, obesity, febrile illnesses in pregnancy, alcohol intake and viral infections have been reported as maternal-environmental risk factors for the development of CHD [10]. The exact mechanisms by which these factors cause CHD remain unknown.

At least 18 distinct types of congenital heart defects are recognized; among these aortic valve stenosis (AVS) is one of the most common and most serious valve disease that has strong genetic basis [11, 12]. Congenital AVS, defined as incomplete obstruction of the valve orifice, is an important category of structural heart defect, and occurs in 3–6% of such cases [13]. There is variability in both the site of obstruction and severity of the obstruction. Sites of obstruction are sub-classified as valvular, subvalvular and supravalvular [14]. About half of infants with severe AVS require surgery [15]. Mild aortic stenosis is difficult to detect prenatally; however, critical aortic stenosis can lead to left ventricular myocardial dysfunction with endocardial fibroelastosis, left atrial dilation and narrowing of the aortic root [16]. These changes can be a prelude to the development of hypoplastic left heart syndrome. The present standard for prenatal screening is mostly based the use of ultrasonography and there is no reliable biologic screening marker for any type of CHD [17].

Recently, some studies have identified an association between dietary folate supplementation and decreased CHD risk [18]. Deficiency in folate is known to result in hyperhomocysteinemia, another risk factor for CHD development [19]. Folate is a B-vitamin that supplies the methyl group for DNA methylation and for other methylation reactions in the cell. Such findings are consistent with a role for epigenetic DNA methylation in the development of CHD. In a recently conducted genome-wide DNA-methylation analysis, we reported evidence of significantly altered CpG methylation levels genome-wide in multiple categories of CHD compared to controls. The study included cases of hypoplastic left heart syndrome, Teratology of Fallot, ventricular septal defect, atrial septal defect and coarctation of the aorta [20].

The prenatal and newborn detection of CHD remains a significant challenge [21, 22]. We undertook a study to examine genome-wide DNA methylation patterns in newborns with AVS to identify genomic regions containing disease-related genes and epigenetic changes that may contribute to CHD pathophysiology. An important objective of the study was to identify DNA methylation biomarkers, serum molecules that could potentially be used in the future for risk estimation and detection of AVS.

## Materials and Methods

Genomic DNA was obtained from neonatal dried blood spots using commercial DNA extraction kits (Qiagen QIAamp^®^) according to manufacturer’s protocol. Many studies have reported genome-wide DNA methylation profiles from archived dried blood spots using the Infinium HumanMethylation450 BeadChip as a suitable template [23, 24]. Blood spot specimens were collected previously for the mandated newborn screening and treatment program run by the Michigan Department of Community Health in the State of Michigan (MDCH). All specimens were collected between 24 and 79 hours after birth. Parents/legal guardians were aware at the time of blood collection that residual blood spots after clinical testing may be utilized for research pending review of such study requests by the MDCH. This study was approved by both the institutional review boards from William Beaumont Hospital and the MDCH. Limited demographic information was available for each subject including date of sample collection, maternal age and race, gestational age at delivery and newborn sex along with the type of CHD anomaly. Suspected or diagnosis-unknown AVS cases were excluded. Unaffected controls had no reported medical disorder and were matched for birth weight, gestational age at delivery, ethnicity, year of birth, and interval from specimen collection to testing. Our cohort included 24 AVS subjects and 24 controls. All specimens were de-identified by removal of further protected health information and researchers were masked to subject identity. Details of the case control cohort are available in S1 Table.

### Genome-wide methylation analysis using the HumanMethylation450

Genome-wide methylation analysis was performed for 48 individuals (24 AVS subjects and 24 controls) using the HumanMethylation450, Illumina’s newest Infinium® HD BeadChip assay for methylation (Illumina, Inc., California, USA), which contains 485,577 methylation sites and requires only 500 ng of genomic DNA. These sites are equally distributed in the genome and represent 96% of RefSeq genes, 95% of CpG islands and an average of 17 CpG sites per covered gene region including the promoter, 5’UTR, coding, and 3’UTR regions. DNA methylation profiling using Illumina Infinium technology with peripheral blood lymphocytes has been used to identify CpG sites associated with disease states [25, 26].

The DNA samples were bisulfite converted using the EZ DNA Methylation-Direct Kit (Zymo Research, Orange, CA) according to the manufacturer´s protocol. The fluorescently stained BeadChips were imaged by the Illumina iScan. Prior to detailed bioinformatic and statistical analysis, data preprocessing and quality control was performed including examination of the background signal intensity of both affected and negative controls, the methylated and unmethylated signals, and ratio of the methylated and unmethylated signal intensities. The processing is done fully according to manufacturer's protocol and 99% of the CpG loci are determined unequivocally.

### Statistical and Bioinformatic analysis

Genome-wide, gene-specific DNA methylation was measured using the Genome Studio methylation analysis package (Illumina). Following the pre-processing described above, a DNA methylation ß-value was assigned to each CpG site. Differential methylation was assessed by comparing the ß-values per individual nucleotide at each CpG site between AVS subjects and controls. In order to avoid potential confounding factors, probes associated with sex chromosomes and/or containing SNPs in the probe sequence (listing dbSNP entries near or within the probe sequence, i.e., within 10 bp of the CpG site) were excluded from further analysis [27–29]. Probes targeting CpG loci associated with SNPs near or within the probe sequence may influence corresponding methylated probes [30]. The remaining CpG sites were taken forward for analysis.

**Fig 1:**
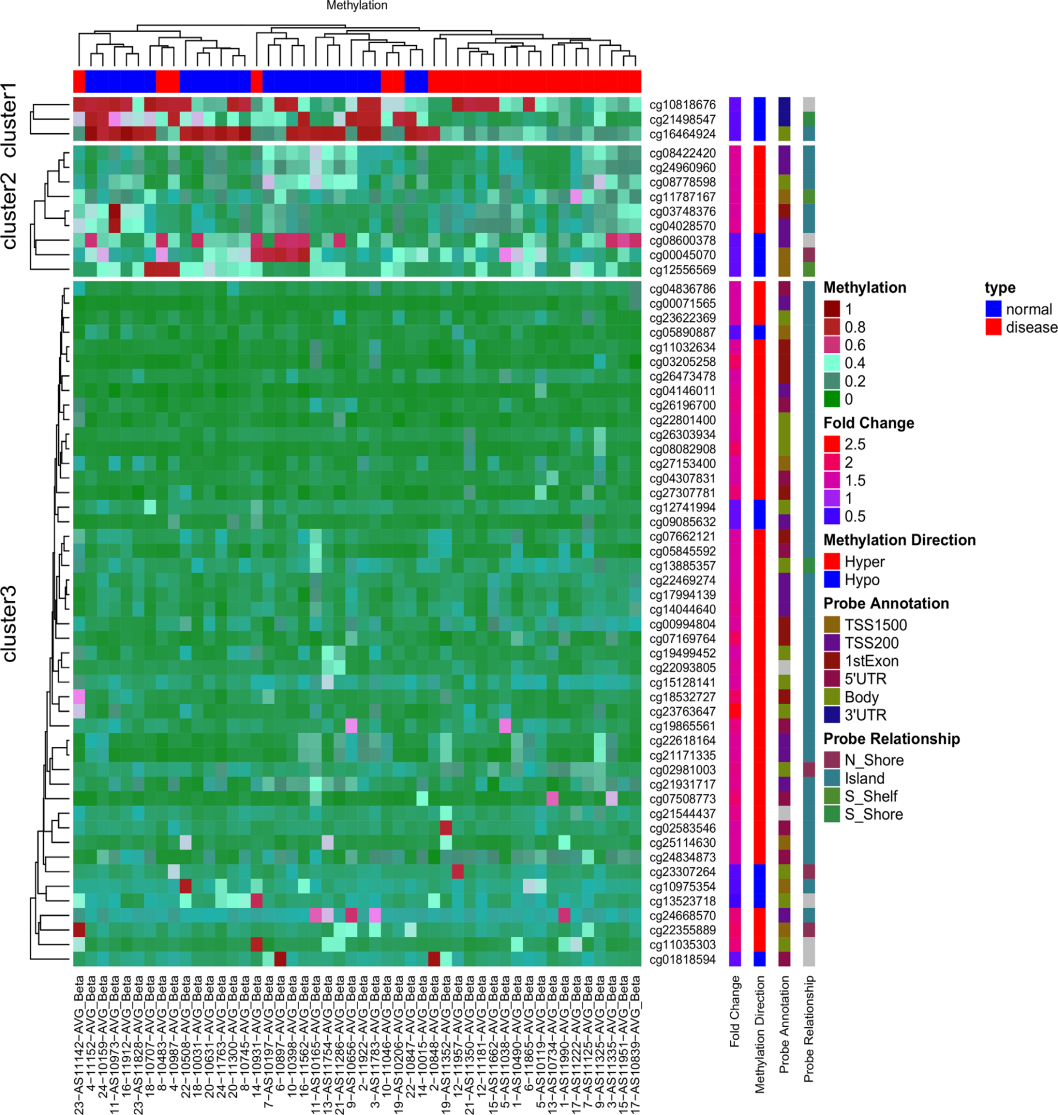
Heatmap of unsupervised hierarchical clustering of AVS data on the basis of 59 differentially methylated CpG sites. Unsupervised hierarchical clustering analysis is very popular in identifying methylation-defined patient sub-groups. Fig 1 displays CpG sites that are at least either 1.5 fold demethylated or 1.5 fold hypermethylated in the disease (AVS) condition (red colored squares) compared to normal subjects (blue colored squares). Differentially methylated CpG sites have been displayed in three clusters (row-wise). The figure also displays direction, fold change in disease, probe relationship and probe annotation of differentially methylated CpG sites. Red color in the heatmap indicates hyper DNA-methylation, and blue hypo DNA-methylation values.

The most discriminating 59 CpG sites were selected based on the pre-set cutoff criteria of >1.5-fold increase and/or >1.5-fold decrease with *p* < 0.05. These sites include the highest 46 hypermethylated and top 13 demethylated CpG sites for further analysis. The identified genes are listed in Table 1. The 59 CpG sites, corresponding to 52 genes, were differentially methylated either in the coding and/or promoter regions and were subsequently used to generate a heatmap using the ComplexHeatmap (v1.6.0) R package (v3.2.2). We have used ward distance for the hierarchical clustering of samples [31] (Fig 1). Subsequently these 59 CpG sites were used to calculate receiver operating characteristic (ROC) curves and the area under the ROC curves (ROC AUC).

**Table 1:** Highly Differentially Methylated CpG sites in Aortic Stenosis subjects. Differentially methylated genes with Target ID, Gene ID, chromosome location, % methylation change and FDR p-value for each gene methylated. CpG sites with significant FDR p-value indicating methylation status and ROC AUC ≥0.75 appear to have a strong potential as diagnostic biomarkers for AVS.

	Target ID	Gene Sym	Chr	% m Change	FDR p-value	AUC
1	cg10818676	DUSP27	1	37.77	6.98E-28	0.72
2	cg21498547	DLGAP2	8	24.36	5.50E-25	0.70
3	cg16464924	GAA	17	38.33	6.17E-27	0.75
4	cg08422420	SDHAP3	5	19.69	2.19E-10	0.67
5	cg24960960	SDHAP3	5	16.57	1.00E-07	0.66
6	cg08778598	SDHAP3	5	22.18	1.67E-09	0.64
7	cg11787167	NPAS3	14	21.09	2.90E-10	0.67
8	cg03748376	OR2L13	1	21.70	6.75E-10	0.61
9	cg04028570	OR2L13	1	21.63	5.12E-13	0.68
10	cg08600378	PRHOXNB	13	22.86	3.02E-19	0.73
11	cg00045070	PCSK9	1	21.65	7.98E-16	0.67
12	cg12556569	APOA5	11	18.95	2.17E-14	0.65
13	cg04836786	HLTF	3	5.68	0.1640	0.76
14	cg00071565	ODC1	2	2.98	0.5085	0.64
15	cg23622369	HSD17B1	17	4.47	0.3173	0.60
16	cg05890887	RPL9	4	2.47	0.2188	0.80
17	cg11032634	TXNRD2	22	4.11	0.3061	0.62
18	cg03205258	TXNRD2	22	3.83	0.1066	0.60
19	cg26473478	C6orf136	6	5.64	0.1647	0.69
20	cg04146011	FBXL6	8	3.07	0.4184	0.55
21	cg26196700	SORD	15	4.62	0.1051	0.75
22	cg22801400	HECTD2	10	3.96	0.3084	0.53
23	cg26303934	C7orf50	7	6.06	0.0640	0.61
24	cg08082908	C7orf50	7	4.27	0.0605	0.64
25	cg27153400	ISOC2	19	5.76	0.1693	0.61
26	cg04307831	CNST	1	5.14	0.2136	0.59
27	cg27307781	CBR1	21	5.43	0.0177	0.53
28	cg12741994	CLDN11	3	4.95	0.0325	0.61
29	cg09085632	PPP2R1B	11	3.42	0.1617	0.52
30	cg07662121	MPV17L	16	8.32	0.0123	0.62
31	cg05845592	SULT1A1	16	5.79	0.1174	0.50
32	cg13885357	KRT3	12	9.17	0.0112	0.68
33	cg22469274	HOXA6	7	6.79	0.0543	0.76
34	cg17994139	HOXA6	7	6.21	0.0519	0.70
35	cg14044640	HOXA6	7	7.56	0.0033	0.69
36	cg00994804	RUNX1	21	9.74	0.0013	0.76
37	cg07169764	MCM6	2	6.86	0.0003	0.51
38	cg19499452	PACS2	14	7.86	0.0345	0.67
39	cg22093805		4	7.03	0.0158	0.51
40	cg15128141	GALNT9	12	10.81	0.0009	0.71
41	cg18532727	C17orf51	17	7.99	2.15E-05	0.61
42	cg23763647	AKR1E2	10	8.50	1.01E-09	0.74
43	cg19865561	MICB	6	9.33	5.63E-05	0.60
44	cg22618164	WDR66	12	10.79	0.0003	0.55
45	cg21171335	WDR66	12	9.91	0.0005	0.51
46	cg02981003	GPR123	10	13.27	6.83E-08	0.57
47	cg21931717	SDHAP3	5	14.60	4.22E-08	0.68
48	cg07508773	WDSUB1	2	7.56	0.0002	0.51
49	cg21544437		2	9.72	0.0001	0.69
50	cg02583546	C14orf4	14	8.76	0.0085	0.54
51	cg25114630	CHSY1	15	8.36	0.0089	0.54
52	cg24834873	ANKRD34B	5	16.61	2.35E-07	0.69
53	cg23307264	KHSRP	19	6.24	1.61E-05	0.60
54	cg10975354	VPS13A	9	9.10	1.15E-10	0.67
55	cg13523718	PTPRN2	7	8.36	5.62E-15	0.68
56	cg24668570	KNDC1	10	21.19	1.30E-37	0.70
57	cg22355889	ELMOD1	11	15.01	1.36E-13	0.55
58	cg11035303	ANO10	3	12.95	4.43E-09	0.61
59	cg01818594	AIMP1	4	9.31	9.29E-06	0.61

### Validation of methylation status by bisulfite sequencing

To confirm the results obtained from the Illumina HumanMethylation450 arrays, we performed pyrosequencing analysis of bisulphite-converted DNA with appropriate oligos using the PyroMark Q24 System and advanced CpG Reagents (Qiagen ^®^).

The p-value for methylation differences between case and normal groups at each locus was calculated as previously described [32]. Filtering criteria for p-values were set at <0.05 and also <0.01 in order to identify the most differentiating cytosines. P-values were calculated with and without Benjamini and Hochberg False Discovery Rate (FDR) correction for multiple testing [33]. The Benjamini and Hochberg correction tolerates more false positive genes than the Bonferroni correction.

Further analysis of the differentially methylated genes was conducted for potential biological significance. ROC curves and ROC AUC were calculated to determine the diagnostic accuracy of specific cytosine loci to differentiate AVS from control groups. Data were normalized using the Controls Normalization Method.

### Gene ontology analysis and functional enrichment

The genes found to be differentially methylated (at FDR p-value < 0.01) were uploaded to the web-based functional annotation tool DAVID V67 (DAVID/EASE, WebGestalt) for Gene Ontology analysis [34, 35] including gene ID conversion, bio-pathways analysis, and the molecular functions of methylated and unmethylated regions. Literature data mining for co-occurrence of gene names and keywords of interest was performed using Chilibot. Only genes for which Entrez identifiers were available were further analyzed. Pathway analysis was carried out using Ingenuity pathway analysis (Ingenuity Systems). Over-represented canonical pathways, biological processes and molecular processes were identified.

### Web Resources

The URLs for data presented herein are as follows:

Illumina: http://www.illumina.com/

Genome Studio: http://www.solexa.com/gsp/genomestudio_software.ilmn

Ensemble: http://www.ensembl.org/

UCSC: http://genome.ucsc.edu/

NCBI: http://www.ncbi.nih.gov/

DAVID/EASE: http://david.abcc.ncifcrf.gov/

Web Gestalt: http://genereg.ornl.gov/webgestalt/

Chilibot: www.chilibot.net

Ingenuity Systems: www.ingenuity.com

Online Mendelian Inheritance in Man (OMIM): http://www.ncbi.nlm.nih.gov/Omim/

## Results

There were no differences in gestational age at birth in weeks: mean (SD) 38.75 (1.42) in AVS subjects vs. 38.88 (1.19) in controls (p = 0.743), nor in the timing of specimen collection after birth (in hours): mean (SD) 31.042 (11.86) in AVS subjects vs. 32.46 (8.62) in controls (p = 0.638). There were no variations in maternal age: mean (SD) 29.87 (4.56) in AVS subjects vs. 29.87 (4.56) years in controls (p-value 1.00). Finally, maternal race and newborn gender were matched for analysis. Of the 52 genes identified during genome-wide methylation analysis, hierarchical clustering analysis demonstrated ~10 as novel principal candidate genes that are commonly methylated and whose methylation was associated with altered gene expression in AVS individuals. Table 1 includes the most significantly differentially methylated CpG sites based on FDR-corrected p-values. The methylation status is represented as percentage methylation for a given probe in the sample. A positive ‘% m Change’ value indicates an average increase in methylation status in AVS subjects compared to control samples. Similarly, a negative ‘% m Change’ value indicates a decrease in methylation status in AVS subjects compared to controls. The p-value indicates the significance of the differential methylation levels. The University of California Santa Cruz (UCSC) gene name and genomic location of the C in the CG dinucleotide and the chromosome on which it is located as provided by Illumina are also shown in Table 1. The results obtained from the DAVID Pathway and Gene Ontology over-representation analysis for canonical pathways and for biological processes are presented in Table 2. Gene Set Enrichment analysis using multiple computational tools showed no significant functional enrichment due to the relatively small size of the gene list. Therefore Gene Ontology information for all genes given in the list was obtained and classified. DAVID pathway analysis software was used to identify molecular pathways associated with genes having differentially methylated CpG sites between AVS subjects and controls. Analysis was done on genes with at least one differentially methylated CpG site based on the uncorrected p-values.

**Table 2:** Over-represented Gene Ontology Molecular Function and Biological Process categories as determined using DAVID Categories: AVS. Biological Process and Metabolic Function categories for over-represented pathways determined using DAVID Pathway and Gene Ontology analysis

Category	Term	Term Description	# of hypo and hyper methylated genes annotated to the term	%of hypo and hyper methylated genes annotated to the term	p-Value	Genes
Biological Process	GO:0048260	Positive regulation of receptor -mediated endocytosis	2	4.17	0.02	APOA5 PCSK9
Biological Process	GO:0048259	Regulation of receptor -mediated endocytosis	2	4.17	0.04	APOA5 PCSK9
Molecular Function	GO:0042802	Identical protein binding	6	12.5	0.02	AIMP1 PCSK9 TXNRD2 CLDN11 RUNX1 MCM6
Molecular Function	GO:0050750	low-density lipoprotein receptor binding	2	4.17	0.03	APOA5 PCSK9
Molecular Function	GO:0070325	lipoprotein receptor binding	2	4.17	0.04	APOA5 PCSK9

**Fig 2:**
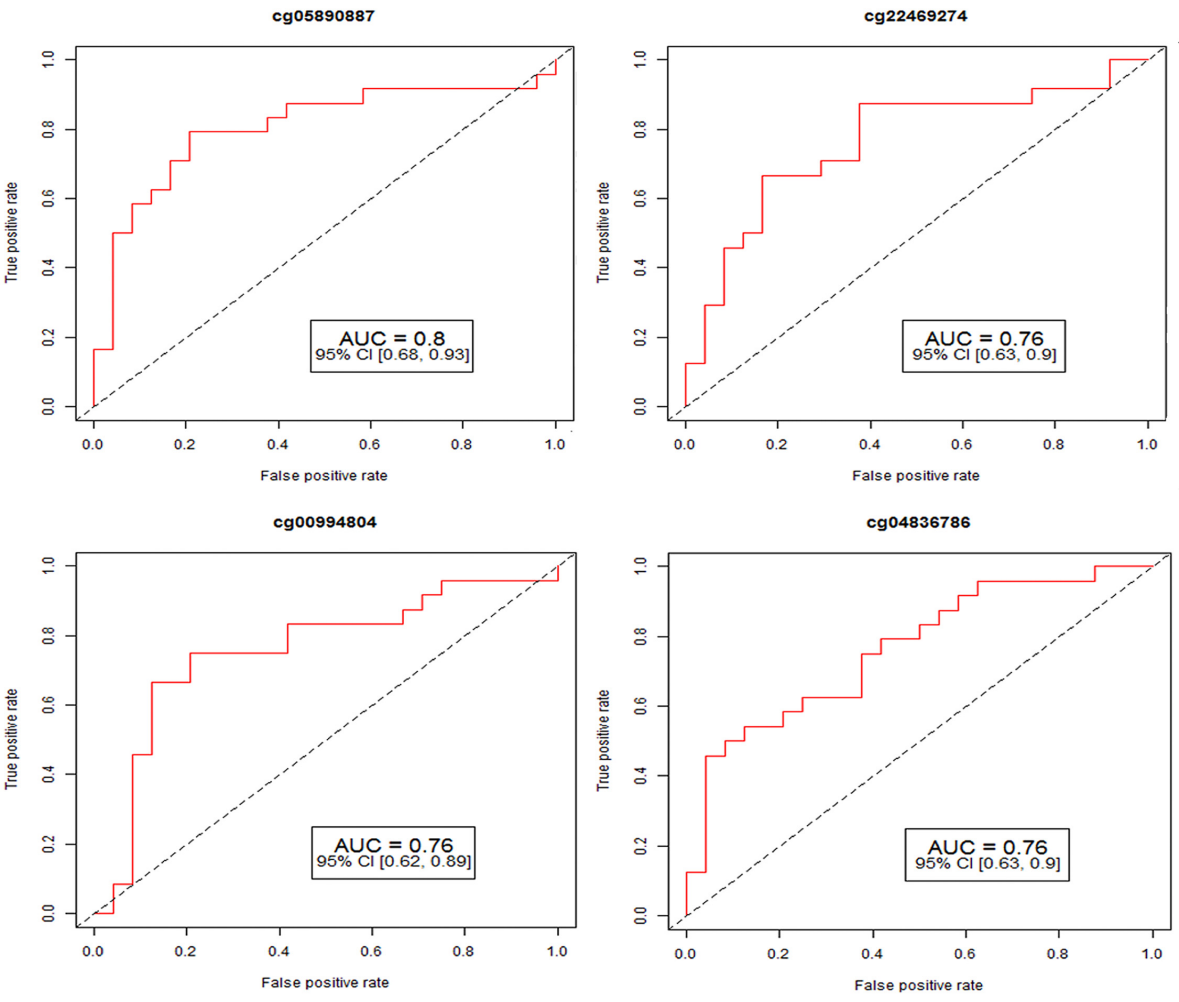
Receiver operating characteristic (ROC) curve analysis of methylation profiles for four specific markers associated with aortic valve stenosis. AUC: Area Under Curve; 95% CI: 95% Confidence Interval. Lower and upper confidence interval was given in parentheses. We have identified six CpG sites (cg16464924, cg05890887, cg26196700, cg22469274 cg00994804 cg04836786) among 52 differentially methylated genes that have ROC AUC ≥0.75. At each locus, the FDR p-value for methylation difference between AVS subjects and controls was highly significantly different. Due to figure resolution, we have included only for four markers.

In combination with the FDR p-value indicating methylation status, the area under the ROC curves can be used to distinguish AVS subjects from controls. All data cleaning and analysis was performed using R (version 3.2.3) and RStudio (version 0.99.489). The CpG sites corresponding to the 52 differentially methylated genes have a ROC AUC ≥0.50 including six CpG sites with ROC AUC ≥0.75: cg16464924 (AUC 075; 95% CI, 0.62 to 0.89), cg05890887 (AUC 0.80; 95% CI, 0.68 to 0.93), cg26196700 (AUC 0.75; 95% CI, 061 to 081), cg22469274 (AUC 0.76; 95% CI, 0.63 to 0.90), cg00994804 (AUC 0.76; 0.62 to 0.89), cg04836786 (AUC 0.76; 0.63 to 0.90). At each locus, the FDR p-value for methylation difference between AVS subjects and controls was highly significantly different. ROC curve analysis (Fig 2) narrowed down the number of markers commonly methylated in AVS for the development and further consideration of their role and validation and also possible use as biomarkers.

Biological processes and molecular function determination for these genes are shown in Table 2. Genes were further grouped according to their Gene Ontology-characterized function. Two genes were identified whose biological processes (*APOA5 PCSK9*) have known molecular function (*AIMP1*, *PCSK9*, *TXNRD2*, *CLDN11*, *RUNX1*, *MCM6*, *APOA5 and PCSK9*).

## Discussion

We have presented genome-wide differential methylation analysis of AVS subjects. To our knowledge, this is the first study that has performed an array-based genome-wide high resolution DNA methylation in a cohort of AVS newborns. We found that epigenetic alteration of CpG sites exhibits a relationship with AVS phenotype, not only of those located within the CpG islands in the promoter region of genes, but also of those distributed throughout the gene including 5’ UTR, coding, and 3’UTR regions. Recent work by our group [20] found a strong association between CpG methylation changes and the presence of multiple different types of CHD. Another recently published study reported DNA methylation changes in patients with Tetralogy of Fallot (TOF) using MassARRAY-based quantitative methylation analysis [36]. That study was, however, limited to targeted promoter regions. Taken together, these studies provide evidence that DNA methylation may be critical in the genetic and environmental interactions underlying cardiac morphogenesis. We have identified 59 CpG sites based on either at least 1.5 fold demethylation or 1.5 fold hypermethylation in disease samples. We hypothesize from the present study that altered expression of one or a combination of the 52 genes corresponding to the 59 CpG sites identified by differential methylation analysis is likely to be responsible for AVS.

There is a well-established and known association between defects in receptor-mediated endocytosis and many diseases such as hypercholesterolemia, a condition characterized by very high levels of cholesterol in the blood. Higher levels of total cholesterol increase the risk of cardiovascular disease [37, 38] including aortic valve stenosis [39] and a high risk for heart disease even in childhood. The report by Chui et al [40] also showed significantly higher serum total cholesterol concentrations in patients with aortic stenosis than in controls. However, molecular mechanisms responsible for the association of hypercholesterolemia with CHD in offspring remain unknown. In the present study, the identification of multiple genes modified in AVS indicates that the phenotype is a complex trait.

*DUSP27* (Dual Specificity Phosphatase 27 (Putative)) is a protein coding gene that belongs to the dual-specificity phosphatase (*DUSP*s) family of proteins. These proteins play important roles in a number of cell types and events, but are preferentially expressed in the heart and skeletal muscles. Li [41] conducted a *Dusp27* knockdown experiment using siRNA to study its effect on myogenic differentiation. These experiments identified this gene’s important role during muscle and heart development. Together, these findings indicate that *Dusp27* may have a more important function in these two tissues. Arrington et al. [42] conducted exome sequencing of DNA samples from multiple affected family members with diverse CHDs and identified gene variations in *DUSP27* among the 18 variations they noted.

*APOA5* (OMIM 606368) or apolipoprotein A5 is associated with plasma triglyceride levels, a major risk factor for coronary heart disease. Earlier association studies were conducted to examine the links between high-density lipoprotein genetics of various genes, including the *APOA5* gene, and aortic valve stenosis risk [43]. However due to limited sample size, their study did not yield strong associations. A significant correlation between the *APOA5* gene polymorphism and the levels of plasma high-density lipoprotein-cholosteal and increased risk for cardiovascular disease was previously identified [44, 45].

Another important gene identified by Gene Ontology analysis is proprotein convertase, subtilisin/Kexin-Type 9 (*PCSK9*, OMIM 607786). *PCSK9* is a serine protease that reduces both hepatic and extrahepatic low-density lipoprotein (LDL) receptor levels and increases plasma LDL cholesterol. Mutation in this gene leads to an increase in the LDL cholesterol level. Cohen et al [46] studied a large population and identified *PCSK9* gene variations associated with differing plasma levels of LDL cholesterol and the risk of coronary heart disease, including myocardial infarction and mortality from coronary disease.

Two other genes displaying altered methylation are Runt-related transcription factor 1 (*RUNX1*; OMIM 151385) on chromosome 21q22.12, and Thioredoxin reductase 2 (*TXNRD2*; OMIM 606448) on chromosome 22q11.21. Deletion of chromosome 21q21.1–22.12 including the *RUNX1* gene has been associated with multiple congenital anomalies and congenital heart defects including aortic stenosis [47, 48]. Thioredoxin reductase is directly involved in reducing proteins such as insulin. Experiments in mice demonstrated *TrxR2* is required for proper heart development [49]. Chromosome micro-deletions (del22q11) involving *TXNRD2* have also been associated with various congenital heart defects including aortic valve stenosis [50]. Helicase-Like transcription factor (*HLTF*; OMIM 603257), a gene that maps to chromosome 3, also plays an important role in the mouse heart development and function [51].

A number of other unrecognized genes identified in the genome-wide analysis are also potentially implicated in the pathogenesis of AVS, however the association between these genes and heart defects and putative mechanisms of action remain to be further explored.

In summary, we have identified alterations in DNA methylation in a number of genes involved in biological processes important to cardiac development in newborns with AVS, supporting the hypothesis that epigenetic modification may play a crucial role in the development of congenital heart defects such as AVS.

In the present study we have identified novel DNA-methylated genes and critical biological pathways that correlate with human aortic valve stenosis. We have demonstrated profound methylation differences in multiple CpG sites in these genes in AVS subjects. The methylation levels of individual CpG sites were used to calculate the area under the ROC curves as a measure of the accuracy of a putative diagnostic test with 59 CpG sites. Many of the identified CpG sites have a higher ROC AUC than 0.5; among them, the six CpGs with the highest AUC of more than 0.75 display strong diagnostic potential. Our overall results raise the possibility of using a large number of different marker combinations for effective detection of AVS. The results suggest that DNA methylation analysis is a molecular technique that might have the potential to be developed into a test for screening and diagnosis of AVS.

Finally the present study also determined that among 52 genes identified, many have not previously been reported in association with AVS. These results also suggest that the selected genes may be involved in the development of AVS by DNA methylation. The functional role of these genes or their potential as novel DNA methylation markers remains to be examined. An important limitation of the present study is that the findings are based on a relatively small number of subjects; however, these results can form the basis for specific hypotheses regarding AVS pathophysiology. Future larger studies based on this preliminary data may contain further clues for new approaches to non-invasive prenatal diagnosis and even prevention of this serious congenital heart malformation.

## Supporting Information

S1 Table Details of the AVS subject cohort and controls used in the present analysis (online only). (DOCX)
