# Supplementary material for: Genome-Wide DNA Methylation Analysis and Epigenetic Variations Associated with Congenital Aortic Valve Stenosis (AVS)
Source: PLoS One. 2016 May 6;11(5):e0154010. doi: 10.1371/journal.pone.0154010 (PMC4859473; doi:10.1371/journal.pone.0154010)
Supplement: S1 Table — (DOCX) [file pone.0154010.s001.docx]

**Table 1 Supplementary**

| **S. No** | **Sex** | **Mom** | | **Gestational  age at birth**  **(weeks)** | **Age at  Collection (hours)** | **status** |
| --- | --- | --- | --- | --- | --- | --- |
|  |  | **Age**  **(years)** | **Race** |  |  |  |
| 1 | male | 21 | white | 38 | 40 | Control |
| 2 | male | 28 | white | 39 | 26 | Control |
| 3 | male | 29 | white | 37 | 26 | Control |
| 4 | male | 18 | white | 40 | 24 | Control |
| 5 | female | 33 | white | 40 | 36 | Control |
| 6 | male | 30 | white | 37 | 44 | Control |
| 7 | female | 31 | black | 40 | 24 | Control |
| 8 | male | 27 | white | 40 | 24 | Control |
| 9 | male | 29 | white | 38 | 24 | Control |
| 10 | male | 33 | black | 40 | 24 | Control |
| 11 | male | 25 | white | 40 | 79 | Control |
| 12 | male | 30 | black | 38 | 25 | Control |
| 13 | male | 38 | white | 38 | 29 | Control |
| 14 | male | 36 | white | 38 | 36 | Control |
| 15 | male | 31 | white | 40 | 25 | Control |
| 16 | male | 31 | white | 39 | 29 | Control |
| 17 | male | 34 | white | 37 | 36 | Control |
| 18 | male | 28 | white | 39 | 36 | Control |
| 19 | male | 31 | white | 38 | 24 | Control |
| 20 | male | 33 | white | 39 | 25 | Control |
| 21 | female | 37 | white | 38 | 34 | Control |
| 22 | female | 27 | white | 40 | 27 | Control |
| 23 | male | 28 | black | 40 | 24 | Control |
| 24 | female | 29 | white | 41 | 24 | Control |
| 1 | male | 21 | white | 37 | 37 | Case |
| 2 | male | 28 | white | 39 | 32 | Case |
| 3 | male | 29 | white | 40 | 24 | Case |
| 4 | male | 18 | white | 38 | 36 | Case |
| 5 | female | 33 | white | 40 | 28 | Case |
| 6 | male | 30 | white | 35 | 38 | Case |
| 7 | female | 31 | black | 39 | 30 | Case |
| 8 | male | 27 | white | 40 | 34 | Case |
| 9 | male | 29 | white | 38 | 30 | Case |
| 10 | male | 33 | black | 37 | 24 | Case |
| 11 | male | 25 | white | 40 | 66 | Case |
| 12 | male | 30 | black | 39 | 36 | Case |
| 13 | male | 38 | white | 40 | 34 | Case |
| 14 | male | 36 | white | 39 | 36 | Case |
| 15 | male | 31 | white | 38 | 24 | Case |
| 16 | male | 31 | white | 38 | 36 | Case |
| 17 | male | 34 | white | 39 | 28 | Case |
| 18 | male | 28 | white | 40 | 24 | Case |
| 19 | male | 31 | white | 39 | 25 | Case |
| 20 | male | 33 | white | 39 | 26 | Case |
| 21 | male | 37 | white | 41 | 35 | Case |
| 22 | female | 27 | white | 40 | 30 | Case |
| 23 | male | 28 | black | 36 | 37 | Case |
| 24 | female | 29 | white | 39 | 29 | Case |
